# Muscle Infarction Following Rapid Glycemic Control in a Patient With Diabetes-Associated Microvascular Disease

**DOI:** 10.7759/cureus.17182

**Published:** 2021-08-14

**Authors:** Leonor Boavida, Joana Carvalho, Susana Oliveira, José Delgado Alves

**Affiliations:** 1 Department of Internal Medicine IV, Hospital Professor Doutor Fernando Fonseca, Amadora, PRT; 2 Department of Internal Medicine IV, Systemic Immune-Mediated Diseases Unit (UDIMS), Hospital Professor Doutor Fernando Fonseca, Amadora, PRT

**Keywords:** diabetes mellitus, insulin treatment, diabetic microvascular complications, muscular infarction

## Abstract

We report a case of a 40-year-old African male with a history of diabetes mellitus with multiple microvascular complications, having recently initiated insulin treatment with a rapid decline in glycosylated hemoglobin (HbA1c) concentration. The patient presented with a sudden onset of right thigh pain and swelling not associated with trauma. Blood work revealed elevated inflammatory markers. A presumptive diagnosis of pyomyositis was made and the patient was treated with intravenous antibiotics with no improvement. Diabetic muscle infarction was then considered and confirmed by magnetic resonance imaging of the affected thigh. As with retinopathy and neuropathy deterioration that have been described as secondary to an aggressive glycemic control, it is possible that muscle myonecrosis may have been consequent to the rapid HbA1c normalization.

## Introduction

Diabetic muscle infarction, or myonecrosis, is a rare, and likely under-recognized, microangiopathic complication of both type 1 and type 2 diabetes mellitus. It is often associated with long-standing history of diabetes, poor metabolic control, and other microvascular complications [1,2]. 

Here we report the uncommon case of a 40-year-old male who presented with diabetic myonecrosis, following the start of insulin treatment and consequent glycemic control.

## Case presentation

A 40-year-old African male with a five-year history of uncontrolled diabetes mellitus with multiple microvascular complications including retinopathy and nephropathy was recently started on insulin treatment with a consequent rapid decline of glycosylated hemoglobin (HbA1c), from 11% to 5.8%. Two months after initiating insulin, the patient presented with a sudden onset of pain and swelling in the right thigh with a progressive functional impairment of the right lower limb. There was no history of fever or associated trauma. On hospital admission he had a swollen, painful and erythematous right thigh and was afebrile. There was no muscle weakness. Blood work revealed elevated creatine kinase (1639 U/L) and C-reactive protein (25 mg/dL) as well as leukocytosis (17.8 x 10^9^/L). 

**Figure 1:**
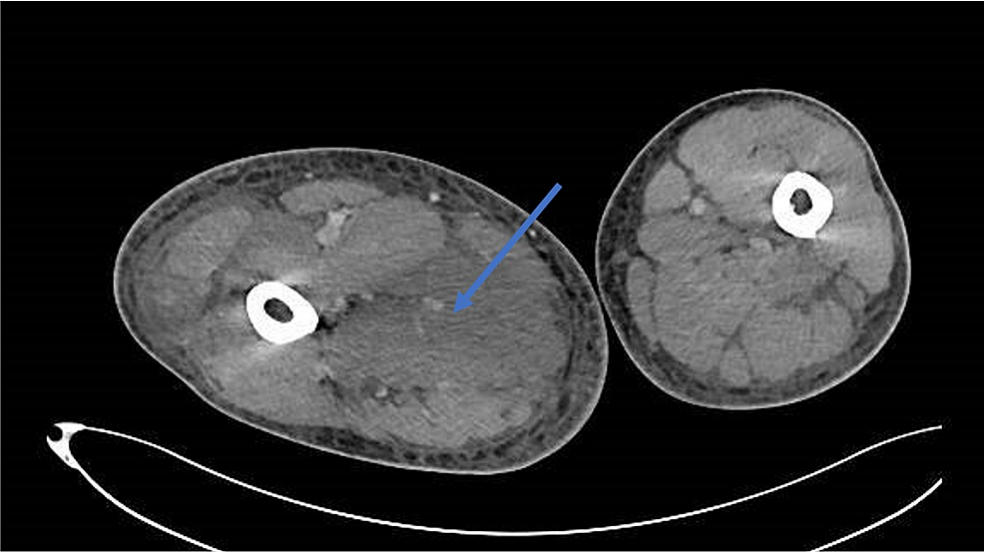
CT scan of the right thigh revealing soft tissue edema involving the muscular planes, particularly in the adductors compartment (arrow) compatible with myositis. CT, computed tomography

Initial work-up included a Doppler ultrasound evaluation that excluded deep vein thrombosis. Computed tomography scan of the right thigh revealed aspects that translated into myositis with soft tissue edema involving the muscular planes, particularly in the adductors compartment (Figure 1).

**Figure 2:**
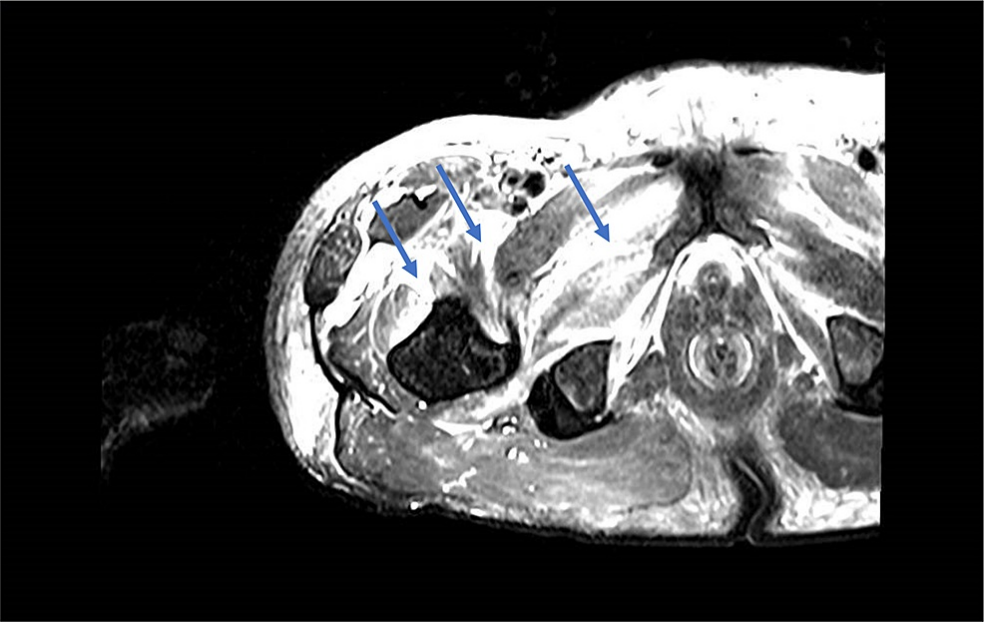
Axial MRI demonstrating increased volume and heterogeneity of the adductor, vastus and sartorius muscles (arrows) associated with perifascial edema and multiple areas compatible with muscle infarction. MRI, magnetic resonance imaging

A presumptive diagnosis of pyomyositis was made and the patient was treated with intravenous antibiotics with no improvement. Extensive laboratory studies were negative for viral, bacterial and parasitic agents. Autoimmunity and toxics were excluded. Diabetic muscle infarction was then considered. Magnetic resonance imaging (MRI) of the affected thigh revealed increased volume and heterogeneity of the adductor, vastus and sartorius muscles associated with perifascial and perimuscular edema and multiple areas of increased contrast caption compatible with muscle infarction (Figure 2). 

**Figure 3:**
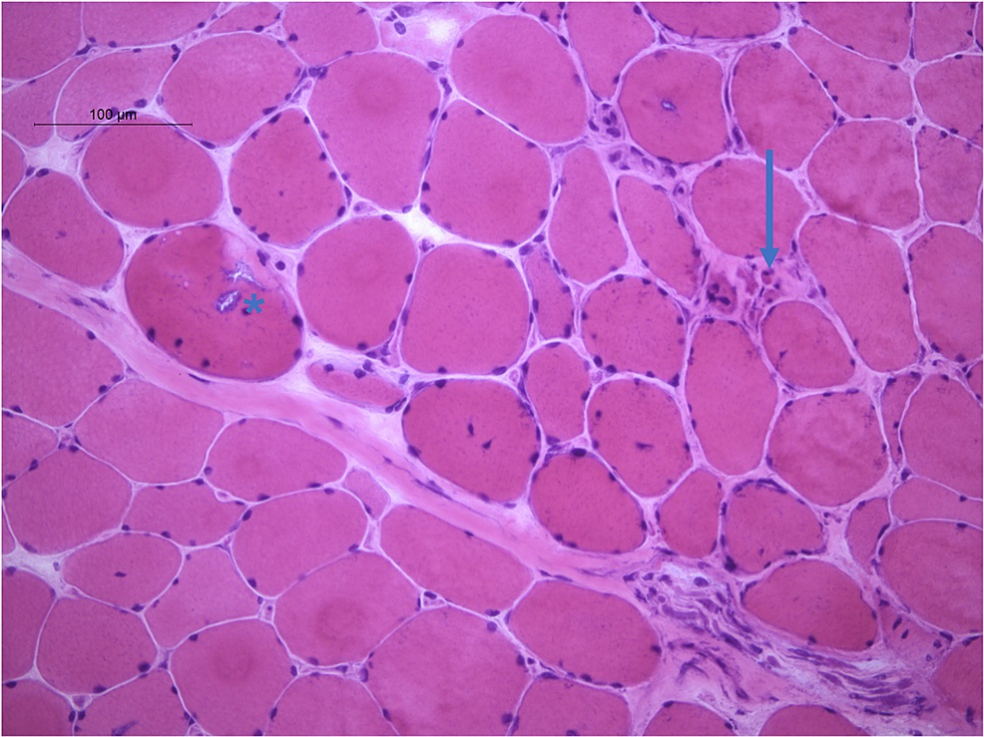
Muscle biopsy under H&E stain showing inflammatory infiltrates (arrow) and focal myonecrosis (asterisk). H&E, hemotoxylin and eosin

Muscle biopsy was obtained revealing inflammatory infiltrate and areas of skeletal muscle necrosis (Figure 3).

These findings supported the diagnosis of diabetic muscle infarction and supportive care was maintained with eventual significant improvement. The patient was discharged after 22 days with an adjusted regimen of insulin treatment and oral antidiabetic drugs.

## Discussion

Diabetic muscle infarction is a rare microvascular complication of diabetes mellitus. It can occur in both type 1 and type 2 diabetes, the majority of patients having various other microvascular complications including retinopathy, nephropathy or neuropathy [1]. Its pathogenesis is uncertain but appears to be related to vascular anomalies, ischemia-reperfusion injury, and alterations in the coagulation-fibrinolysis system associated with longstanding and poorly controlled diabetes [1-3].

Clinical manifestations include acute or subacute onset of muscle pain, swelling and tenderness involving the thigh or calf muscles [1-3]. As this case highlights, awareness of the syndrome is of utmost importance for its characteristic clinical features will frequently suggest the diagnosis in the setting of poorly controlled diabetes mellitus. Consequently, laboratory and imaging studies are aimed mainly at excluding other disorders such as trauma, deep vein thrombosis, hematoma, abscess, fasciitis, inflammatory myopathies, myositis associated with connective tissue diseases, infective myositis, infiltrating neoplasm, rhabdomyolysis and other vascular causes [4]. Blood work often suggests an inflammatory process with leukocytosis, and elevated erythrocyte sedimentation rate and C-reactive protein making the distinction with infection more difficult. Magnetic resonance imaging (MRI) with intravenous contrast enhancement is the diagnostic imaging tool of choice and findings include increased T2 signal in the affected muscle, fascia and subcutaneous tissues [5]. Muscle biopsy is not required for the diagnosis of muscle infarction, being most often reserved for patients with an atypical clinical course. Treatment of diabetic muscle infarction involves symptomatic management with rest, optimal glycemic control, analgesia and low-dose salicylic acid [1].

Rigorous glycemic control has become the standard approach to type 2 diabetes care despite recent evidence showing no meaningful benefit of intensive (compared with moderate) glycemic control for microvascular outcomes [6]. Unintended consequences, besides greater risk of severe hypoglycemia, and higher burden of treatment [6], include the development of microvascular complications related to the rapidity of glycemic improvement [7]. As with neuropathy [7] and retinopathy [8] deterioration that have been described as secondary to aggressive glycemic control, it is possible that muscle myonecrosis was consequent to rapid HbA1c normalization, although further investigation is necessary. Possible mechanisms include a diffuse microvascular process involving inflammation and endothelial dysfunction that can occur in the setting of rapid improvements to glucose control [7]. Nevertheless, as this case suggests, intensive insulin treatment inducing a rapid HbA1c decline should prompt vigilance and caution regarding the risk of microvascular disease complications, particularly in patients with uncontrolled diabetes. Therapeutic goals need to be individualized and contextualized to each patient’s needs.

## Conclusions

We report a rare case of diabetic muscle infarction, with the particularity of having occurred following insulin treatment initiation and possibly as a result of rapid HbA1c normalization. Awareness of the syndrome is fundamental to early recognition, for its diagnosis will often be suggested by typical clinical finding in the setting of longstanding and poorly controlled diabetes. This case highlights the need for caution regarding intensive insulin treatment and the risk of associated microvascular disease complications, particularly in long-term diabetes. 
